# Recent CR1 non-LTR retrotransposon activity in coscoroba reveals an insertion site preference

**DOI:** 10.1186/1471-2164-9-567

**Published:** 2008-11-27

**Authors:** Judy St John, Thomas W Quinn

**Affiliations:** 1 Rocky Mountain Center for Conservation Genetics and Systematics, Division of Natural Sciences and Mathematics, University of Denver, Denver, Colorado, USA

## Abstract

**Background:**

Chicken repeat 1 (CR1) is a taxonomically widespread non-LTR retrotransposon. Insertion site bias, or lack thereof, has not been demonstrated for CR1. Recent CR1 retrotranspositions were used to examine flanking regions for GC content and nucleotide bias at the insertion site.

**Results:**

Elucidation of the exact octomer repeat sequence (TTCTGTGA) allowed for the identification of younger insertion events. The number of octomer repeats associated with a CR1 element increases after insertion with CR1s having one octomer being youngest. These young CR1s are flanked by regions of low GC content (38%). Furthermore, a bias for specific bases within the first four positions at the site of insertion was revealed.

**Conclusion:**

This study focused on those loci where the insertion event has been most recent, as this would tend to minimize noise introduced by post-integration mutational events. Our data suggest that CR1 is not inserting into regions of higher GC content within the coscoroba genome; but rather, preferentially inserting into regions of lower GC content. Furthermore, there appears to be a base preference (TTCT) for the insertion site. The results of this study increase the current level of understanding regarding the elusive CR1 non-LTR retrotransposon.

## Background

Eukaryotic genomes contain a large percentage of highly and moderately repetitive DNA [1]. Included in the moderately repetitive DNA, are transposable elements (TEs). TEs are categorized into two main classes. DNA transposons (class II) are able to self-excise and move to a new location in the genome while retrotransposons (class I) use an RNA intermediate resulting in a transposed copy. Retrotransposons can further be divided into two categories, those possessing long terminal repeats of 250–600 base pairs (bp) termed LTR retrotransposons and those without LTRs (non-LTR retrotransposons). Non-LTR retrotransposons are thought to be the oldest of the retrotransposons, originating at least 500–600 million years ago [2]. It has been suggested that the non-LTR retrotransposons gave rise to eukaryotic LTRs, which in turn gave rise to myriad viruses including the vertebrate retroviruses [3].

**Figure 1:**
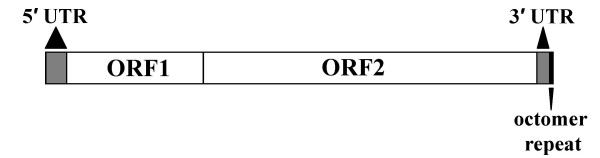
**Graphic representation of the CR1 non-LTR retrotransposon**. The two open reading frames (ORF1 and ORF2) encode proteins necessary for the successful retrotransposition of the element. The 5' untranslated region (UTR) and 3' UTR flank the two ORFs. Located adjacent to the 3' UTR is the octomer repeat (TTCTGTGA)_N_.

Full length (4–6 kb) non-LTR retrotransposons [2], such as L1 and the taxonomically widely distributed chicken repeat 1 (CR1; Fig. 1), contain a 5' untranslated region (UTR), two open reading frames (ORF1 and ORF2) and a 3' UTR [4]. The L1 3' UTR contains a conserved G-rich polypurine motif [4]. Likewise, motifs within the CR1 3' UTR are highly conserved suggesting that this region may act as a recognition site for reverse transcriptase [5]. The CR1 3' UTR also possess one to four copies of an octomer terminal repeat originally described in chicken as NATTCTRT by Silva and Burch [6]. More recently it has been suggested that the CR1 octomer repeat is better represented by a single base shift in the 3' direction; ATTCTRTG [7].

Approximately 200,000 copies of CR1 are found in the chicken genome [7] with 98% of these being less than 2000 bp and most containing less than 1000 bp [8]. Currently it is not known whether the widely dispersed CR1 has preferential insertion sites, whether its distribution is due to selection against deleterious insertions or whether it inserts randomly. Silva and Burch [6] reported the presence of a six base sequence within the preintegration site of the CR1 element considered in their study that contains the last six nucleotides of the octomer NATTCTRT. This sequence homology between the octomer and the preintegration sequence led Silva and Burch [6] to suggest that there exist some sequence preference for CR1 integration.

The occurrence of a CR1 element in the intron of the lactate dehydrogenase B (LDH-B) gene in the waterfowl coscoroba (*Coscoroba coscoroba*) and Cape Barren goose (*Cereopsis novaehollandiae*) has been reported [9]. The corresponding introns in two closely related taxa, the tundra swan (*Cygnus columbianus*) and the snow goose (*Anser caerulescens*) lack this CR1 element suggesting that this insertion took place after the divergence of these species (9–11 million years ago). That makes this CR1 insertion the most recent of those described thus far [9]. Using this relatively young yet highly truncated copy of CR1 (193 bp) as a probe in Southern blot analysis showed that waterfowl genomes possess homologous CR1 elements [9]. Further, the absence of hybridization with the sister order Galliformes suggests that this subfamily of CR1 expanded within waterfowl alone. More recently, several other CR1 insertions in the common ancestor of coscoroba and Cape Barren goose have been discovered (JS and TWQ unpublished data). Such multiple recent inserts provide strong evidence that this CR1 subfamily is actively retrotransposing in the Cape Barren goose/coscoroba lineages. Furthermore, the entire chicken genome has been sequenced and there do not appear to be any active CR1 elements present. Thus, waterfowl provide a unique source of information for recent insertion events of CR1 elements.

The main goal of our study was to determine whether there were any apparent common features in the regions flanking CR1 elements that would indicate insertion site targeting or bias, with a specific focus on the 3' flanking region. Recently retrotransposed CR1 elements allow for the examination of these regions and for determination of any consensus flanking sequence. Sequence homology would indicate the extent to which CR1 insertion occurs at specific sites in the genome. Our study benefits from focusing on those loci where the insertion event has been most recent, as this would tend to minimize noise introduced by post-integration mutational events.

## Results

A total of 145 CR1 inserts were recovered using the rapid capture method [10] and the young CR1 element located in the coscoroba LDH-B gene [9] was added to this dataset resulting in 146 CR1 elements. In 81 cases, cloned CR1s were truncated at a *Csp*6I restriction site located within ORF2 approximately 238 bps from the 3' end of ORF2; presumably due to the earlier use of that restriction enzyme in the rapid capture method [10]. The 146 CR1 elements belong to six distinct subfamilies, with subfamily I exhibiting evidence of recent activity in waterfowl [9,11]. Because of this apparent recent activity the analyses were focused on members of subfamily I (N = 119).

Clarification of the octomer repeat sequence was imperative not only for the correct identification of younger CR1 elements but also for the precise determination of the boundary between CR1 and pre-integration sequences. Recent studies of the chicken genome led the International Chicken Genome Sequencing Consortium (ICGSC) [7] to propose that the frame of the octomer repeat, NATTCTRT [6], should be shifted one base pair in the 3' direction, to become ATTCTRTG. However, in a study of waterfowl, St. John *et al*. [9] aligned homologous sequence from several species with and without a CR1 insert at a particular locus and they observed that the terminal sequence of the insert included an additional adenine at the 3' end such that the insert ended with ATTCTGTGA. This raised some question about whether the octomer repeat frame should have, in fact, been shifted two bases rather than one by the ICGSC. To address this, only those loci with octomer repeat(s) that match the ICGSC definition exactly (ATTCTRTG) were selected from the 119 member subfamily I dataset. Inspection of the nucleotide immediately 3' to the terminal octomer repeat in all resulting 63 loci revealed that all but three (95%) had an adenine at this position. This further supports the idea that the octomer repeat frame should be defined as TTCTRTGA. Additionally, the total number of repeats, either ATTCTRTG or TTCTRTGA, in subfamily I was tallied and both octomer sequences occurred at equal frequencies. These observations supported a frame shift one base to the right from that defined by the ICGSC [7]. Our new definition, TTCTRTGA, was further refined when we observed that the fifth base, originally defined as 'R', was a guanine in 170/178 (96%) of those repeats found in all subfamily I elements. Furthermore, in four of the eight exceptions, there was a pyrimindine, not a purine found at this position. Thus, we defined the octomer repeat sequence as TTCTGTGA.

A set of 39 CR1s from subfamily I contained octomers (TTCTGTGA) that had one or two base substitutions within this region. The 48 base substitutions located across these octomers were not evenly distributed (Table 1). Base substitutions were most frequent at positions seven and eight and least frequent at positions two and four. Furthermore, the mutational spectrum was different for those CR1s that contained more than one repeat, with most substitutions occurring in the terminal octomer (Table 1).

**Table 1:** Number of base substitutions found at each position in octomer repeats.

CR1s^1^	T	T	C	T	G	T	G	A		T	T	C	T	G	T	G	A		T	T	C	T	G	T	G	A
5	0	0	1	1	2	2	0	1																		
27	1	1	0	0	1	1	0	3		2	0	2	0	2	2	12	3									
7	0	0	0	0	0	0	1	0		0	0	1	0	1	0	1	0		1	0	0	0	1	1	2	2

^1^Represents the number of elements that possess 1, 2 or 3 octomer repeats containing 1 or 2 base substitutions.

A total of 60 CR1s from subfamily I were found to contain 1–4 perfect octomer repeats with most having just one or two repeats (Table 2). Three of these sequences contained less then ten bases of 3' flanking region and were eliminated from further analyses. The length of the 3' flanking sequences from the remaining 57 subfamily I CR1s with perfect octomer repeats ranged from 11 to 942 bp, with a mean of 146 bp and a median of 95 bp. Total GC content of the 3' flanking sequences calculated for clones containing at least 50 bp of 3' flanking sequence was 38.2% ± 2.08.

**Table 2:** Number of octomer repeats associated with CR1 elements from subfamily I

Number of repeats^1^	Number of CR1s^2^	Number with perfect repeats^3^	Number with perfect repeats and intact ORF2 and 3' UTR^4^
1	24	19	11
2	61	33	14
3	16	7	3
4	1	1	1

^1^Refers to the number of recognizable octomer repeats (TTCTGTGA) associated with a CR1 excludes those containing more than two base substitutions, indels or truncations.

^2^Represents the number of CR1 elements that contain 1–4 octomer repeats.

^3^Represents the number of CR1 elements that contain perfect repeats (TTCTGTGA).

^4^Represents the number of CR1 elements that contain perfect repeats and intact ORF2s and 3' UTRs.

We noted that the frequencies of the ten bases immediately flanking CR1s with one, two, or three and four perfect repeats and intact ORF2s and 3' UTRs (those ORF2s without stop codons or frame shift mutations and those 3' UTRs without indels) were different (Table 3). In fact, the chi-square goodness of fit test for the first ten bases flanking the 11 CR1s containing one perfect octomer and intact ORF2s and 3' UTRs was highly significant (*X*^2 ^= 39.5; *P *< 0.001) suggesting that the occurrence of specific bases within those first ten flanking positions was not random. For the 14 CR1s containing two perfect octomers and intact ORF2s and 3' UTRs, the chi-square goodness of fit test for the first ten flanking bases was also significant (*X*^2 ^= 22.3; *P *< 0.01). The chi-square goodness of fit test for the first ten bases flanking the CR1s containing three or four perfect octomer repeats, intact ORF2s and 3' UTRs was not significant (*X*^2 ^= 15; *P *> 0.05). However, the sample size for this set was only four. Frequencies of bases at the first three flanking positions in the subset with one perfect octomer and intact ORF2s and 3' UTRs were significantly different from random (*P *< 0.01; Table 3). The bases TTC occurred most frequently at these positions with a T occurring at the forth position with a distribution significantly different from random (*P *< 0.05) in this dataset.

**Table 3:** Observed base occurrence at flanking position 1–10

		position									
	Nucleotide	1	2	3	4	5	6	7	8	9	10
	
1^1^ N = 11	A	1	2	3	2	1	3	3	1	4	3
	T	**10**^3^	**8**	2	7	3	4	4	4	1	4
	C	0	1	**6**	1	5	2	1	4	2	4
	G	0	0	0	1	2	2	3	2	4	0
	
	expected A/T^2^	3.41									
	expected G/C	2.09									
		position									
	Nucleotide	1	2	3	4	5	6	7	8	9	10
	
2 N = 14	A	6	6	2	3	1	2	1	5	7	3
	T	7	5	6	5	6	5	3	5	2	3
	C	1	1	6	3	2	1	**7**	3	1	5
	G	0	2	0	3	5	6	3	1	4	3
	
	expected A/T	4.34									
	expected G/C	2.66									

		position									
	Nucleotide	1	2	3	4	5	6	7	8	9	10
	
3 and 4 N = 4	A	0	3	1	1	1	2	2	1	0	0
	T	3	0	1	3	2	1	1	1	1	2
	C	0	1	1	0	0	1	1	0	2	0
	G	1	0	1	0	1	0	0	2	1	2
	
	expected A/T	1.24									
	expected G/C	0.76									

^1^Indicates the number of octomer repeats in each set of CR1 elements.

^2^Expected nucleotide frequencies were based on a 38% GC content and a 62% AT content.

^3^Bold indicates significant deviation from expected frequencies (*P *< 0.01).

## Discussion

The CR1 elements reported here represent recent insertion events in the coscoroba genome making these elements a logical tool for the investigation into possible insertion site targets or biases for CR1. The exact sequence of the octomer repeat(s) associated with the CR1 3' UTR was initially reported to be NATTCTRT [6] and more recently described as ATTCTRTG [7]. Among coscoroba sequences the octomer is better described as TTCTGTGA. Base substitutions found in octomer repeats suggest that positions seven and eight in the terminal octomer are more vulnerable to base substitutions (Table 1). Base substitutions at positions two and four were extremely rare. Perhaps this pattern is due to the mechanics involved in reverse transcription initiation. Alternatively, mutations could be generated in the terminal octomer through a slippage effect during replication. It does not appear that CR1 elements begin with a defined number of octomer repeats but that the number of octomer repeats increases independently over time after retrotransposition. It is noteworthy that in subfamily I, 79% of the CR1s with one octomer contained a perfect octomer compared to those with two octomer repeats with only 54% being perfect (Table 2). CR1s possessing three octomer repeats followed this pattern with 44% having perfect octomers. Furthermore, the percent of CR1s with perfect octomers and intact ORF2s and 3' UTRs decreased from 46% for those with one octomer to 23% and 19% for those with two and three octomers, respectively (Table 2). The CR1s with perfect octomer repeats and intact ORF2s and 3' UTRs are likely the result of the most recent retrotransposition activity. This suggests that CR1s with one octomer are younger than those with two or more repeats.

Sequence analysis of the flanking regions revealed a non-random distribution of the bases immediately flanking CR1s with one or two perfect octomers and intact ORF2s and 3' UTRs (Table 3). Especially interesting was the discovery that there exists a different base bias between those clones possessing one or two perfect octomers and intact ORF2s and 3' UTRs (Table 3). The first four bases immediately 3' to the octomer from CR1s with one perfect octomer and intact ORF2s and 3' UTRs were most likely TTCT. Those CR1s with two perfect octomer repeats and intact ORF2s and 3' UTRs were equally likely to have an A or T at the first two positions and a T or C at position three (Table 3). Sequence bias at the bases adjacent to the octomer might reflect a preference involving these bases in enzymatic activity related to insertion events [12]. It is also noteworthy that TTCT are the first four bases of the octomer (TTCTGTGA). It is possible that such an insertion site preference for TTCT could provide for slippage to occur; thereby increasing the number of repeats with time. Younger CR1 elements starting with one octomer gain repeats through this slippage mechanism during replication.

Although it appears that CR1 does have some nucleotide bias for insertion the possibility exists that the data could be biased if differential CR1 removal occurs within the genome. For example, the insertion of TEs around centromeres appears to increase the likelihood that they will not be eliminated or inactivated [13]. Heterochromatic DNA contains a much higher density of TEs, containing up to 90 fold more [14]. This could indicate that TEs target the heterochromatin for insertion, that there is a lower rate of deletion in this region, a fixation bias exists in heterochromatin perhaps due to higher selective constraints or that fixation bias is due to positive selection that acts to fix TEs within this region [14]. Interestingly, it has been demonstrated that euchromatic genes that become associated with heterochromatin through chromosomal rearrangement can be transcriptionally silenced [15], which could help to explain a fixation bias to this region based on avoidance of negative selection. Datasets containing younger CR1 elements would be less susceptible to these post-insertion cellular regulation events.

The GC content of the 3' flanking sequence for the coscoroba subfamily I CR1s containing perfect octomer repeats with sequences over 50 bp in length was 38% ± 2.08. Average GC content of vertebrate genomes ranges from 41% for humans to 47.4% for pufferfish [16] with chicken genomes containing an average of 47% GC [16]. Earlier reports suggested that most CR1s were located in the GC rich regions of the chicken genome [17,18]. The data presented here are not consistent with these findings although there may be some bias due to the younger age of the subfamily I elements. Interestingly, the flanking regions of recent L1 insertions contain a higher GC content than older inserts [19] suggesting that L1 preferentially inserts into areas of higher GC content or that they are more frequently deleted in areas of low GC content. It is possible that newer CR1 elements insert throughout the genome and those that are not removed are located in GC rich regions.

## Conclusion

Our data suggest that CR1 is not inserting into regions of higher GC content within the coscoroba genome; but rather, preferentially inserting into regions of lower GC content. Furthermore, there appears to be a base preference (TTCT) for the insertion site. The results of this study increase the current level of understanding regarding non-LTR retrotransposons.

## Materials and methods

### Isolation of CR1 elements

A rapid capture technique that allows for the isolation of specific target sequences from uncharacterized genomes was used to acquire copies of CR1 from coscoroba [10]. Sequencing was performed using a Quickstart kit (Beckman Coulter) following manufacturer's protocol except using half reaction volumes (10 μl). Sequencing reactions were run on the CEQ8000 XL DNA Analysis System (Beckman Coulter) and aligned in Sequencher 4.1.4 (Gene Codes Corporation). CR1 sequences were submitted to the National Center for Biotechnology Information (NCBI) database (GenBank accession numbers EU681026–EU681170).

### Data analysis

Recently inserted CR1s were defined by the possession of perfect octomer repeats and further categorized by having corresponding intact ORF2s and intact 3' UTRs. The 3' flanking sequences from these recently inserted CR1 were evaluated for GC content. To test for randomness in the first 10 positions directly adjacent to the octomer, chi-square goodness of fit test was employed. The binomial distribution was subsequently used to determine which of those first 10 positions, if any, were significantly different from random (*P *< 0.01). Expected frequencies were calculated based on the GC content of the 3' flanking region. The CR1s were tested as one group and then separated into subsets that aimed at classifying the CR1s by age in order to aid in the identification of target site preferences.

## Abbreviations

CR1: chicken repeat 1; LTR: long terminal repeat; ORF: open reading frame; UTR: untranslated region.

## Authors' contributions

JS generated the data, performed the data analyses, and wrote the manuscript. TWQ provided general oversight and assisted in the drafting and editing of the manuscript. This manuscript has been approved by both authors.

## References

[B1] Britten RJ, Kohne DE (1968). Repeated sequences in DNA. Science.

[B2] Kazazian HH (2004). Mobile elements: drivers of genome evolution. Science.

[B3] Burke WD, Malik HS, Rich SM, Eickbush TH (2002). Ancient lineages of non-LTR retrotransposons in the primitive eukaryote, *Giardia lamblia*. Mol Biol Evol.

[B4] Boissinot S, Entezam A, Young L, Munson PJ, Furano AV (2004). The insertional history of an active family of L1 retrotransposons in humans. Genome Res.

[B5] Kajikawa M, Ohshima K, Okada N (1997). Determination of the entire sequence of turtle CR1: the first open reading frame of the turtle CR1 element encodes a protein with a novel zinc finger motif. Mol Biol Evol.

[B6] Silva R, Burch JBE (1989). Evidence that chicken CR1 elements represent a novel family of retroposons. Mol Cell Biol.

[B7] International Chicken Genome Sequencing Consortium (2004). Sequence and comparative analysis of the chicken genome provide unique perspectives on vertebrate evolution. Nature.

[B8] Wicker T, Robertson JS, Schulze SR, Feltus FA, Magrini V, Morrison JA, Mardis ER, Wilson RK, Peterson DG, Paterson AH, Ivarie R (2005). The repetitive landscape of the chicken genome. Genome Res.

[B9] St. John J, Cotter JP, Quinn TW (2005). A recent chicken repeat 1 (CR1) retrotransposition confirms the coscoroba-Cape Barren goose clade. Mol Phylogen Evol.

[B10] St John J, Quinn TW (2008). Rapid capture of DNA targets. BioTechniques.

[B11] St John J, Quinn TW (2008). Identification of novel CR1 subfamilies in an avian order with recently active elements. Mol Phylogenet Evol.

[B12] Jurka J (1997). Sequence patterns indicate an enzymatic involvement in integration of mammalian retroposons. Proc Natl Acad Sci USA.

[B13] Kidwell MG, Lisch DR (1998). Transposons unbound. Nature.

[B14] Blumenstiel JP, Hartl DJ, Lozovsky ER (2002). Patterns of insertion and deletion in contrasting chromatin domains. Mol Biol Evol.

[B15] Carvalho C, Pereira HM, Ferreira J, Pina C, Mendonca D, Rosa AC, Carmo-Fonseca M (2001). Chromosomal G-dark bands determine the spatial organization of centromeric heterochromatin in the nucleus. Mol Biol Cell.

[B16] Flint J, Tufarelli C, Peden J, Clark K, Daniels RJ, Hardison R, Miller W, Philipsen S, Tan-Un KC, McMorrow T, Frampton J, Alter BP, Frischauf A-M, Higgs DR (2001). Comparative genome analysis delimits a chromosomal domain and identifies key regulatory elements in the α globin cluster. Hum Mol Genet.

[B17] Olofsson B, Bernardi G (1983). The distribution of CR1, and Alu-like family of interspersed repeats, in the chicken genome. Biochim Biophys Acta.

[B18] Coullin P, Bed'Hom B, Candelier JJ, Vettese D, Maucolin S, Moulin S, Galkina SA, Bernheim A, Volobouev V (2005). Cytogenetic repartition of chicken CR1 sequences evidenced by PRINS in Galliformes and some other birds. Chromosome Res.

[B19] Ovchinnikov I, Rubin A, Swergold GD (2002). Tracing the LINEs of human evolution. Proc Natl Acad Sci USA.

